# Piriformis-Sparing vs. Conventional Posterior Approach in Total Hip Arthroplasty: A Retrospective Analysis of the Functional Outcomes

**DOI:** 10.3390/medicina61040609

**Published:** 2025-03-27

**Authors:** Müren Mutlu, Hakan Zora, Gökhan Bayrak, Ömer Faruk Bilgen

**Affiliations:** 1 Department of Orthopedics and Traumatology, Private Medicabil Hospital, 16140 Bursa, Türkiye; murenmutlu@hotmail.com (M.M.); ofbilgen@gmail.com (Ö.F.B.); 2 Department of the Physiotherapy and Rehabilitation, Faculty of Health Sciences, Muş Alparslan University, 49250 Muş, Türkiye; fztgokhanbayrak@gmail.com

**Keywords:** hip replacement arthroplasty, hip dislocation, length of stay, postoperative pain

## Abstract

*Background and Objectives:* The posterior approach in total hip arthroplasty (THA) is widely used among surgeons. This study compares dislocation rates and functional outcomes between patients using a piriformis tendon-sparing posterior approach (PSPA) and those using a conventional posterior approach (CPA). *Materials and Methods:* 350 patients who underwent THA between 2016 and 2020 were retrospectively reviewed, with 163 patients receiving a PSPA and 187 receiving a CPA. Dislocation complication and the functional outcomes including the baseline and postoperative sixth-week pain and Oxford Hip Score, sixth-week Ranawat internal rotation test, and sixth-month acetabular inclination and anteversion angle were recorded. Hospital stay and the duration of surgery were also noted. *Results:* Implant dislocation occurred in three (1.6%) patients only in the CPA group at six weeks postoperatively (*p* = 0.104). No differences were noted in surgery time, baseline and postoperative pain, or hip function (*p* < 0.05). The Ranawat internal rotation test was positive in 89.6% of the PSPA group and 40.1% of the CPA group at six weeks (*p* = 0.001). The inclination angle was better in the PSPA group (*p* = 0.001), but there was no difference in anteversion angle (*p* = 0.523) at the sixth month postoperatively. The PSPA group had a shorter hospital stay (mean = 2.14 days) compared to the CPA group (mean = 2.47 days) (*p* = 0.006). *Conclusions:* The absence of dislocation cases in the piriformis-sparing approach suggests that the preservation of the piriformis tendon, especially in the early period, may have reduced the risk of prosthesis dislocation by increasing joint stability from a clinical perspective. Further research is needed to evaluate the long-term impact of the piriformis-sparing posterior approach regarding the dislocation rates and functional outcomes.

## 1. Introduction

Many surgeons prefer the posterior approach in total hip arthroplasty (THA) surgery. The posterior approach was developed by Moore in 1950 and has undergone some subsequent modifications [1]. However, the posterior approach has the main disadvantage of cutting the short external rotator muscles necessary to reach the hip joint [2]. The joint capsule and short external rotator muscles are critical in hip joint stability. The piriformis muscle is a part of the hip external rotators, rotates the femur externally with hip extension, and brings the femur into abduction with hip flexion [3]. Functionally, the piriformis muscle stabilizes the hip by mechanically restricting internal rotation during weight-bearing. The hip’s external rotator and abductor muscles hold the femoral head within the acetabulum in flexion [4].

In the posterior approach of THA, repair of the capsule and external rotators, especially the piriformis muscle, is essential for joint stability [4]. Although experimental and laboratory studies have emphasized the importance of repairing posterior structures, a significant proportion of these ruptures are associated with the force applied during postoperative rehabilitation [4,5]. Therefore, new surgical approaches have been defined to open these muscles at a level that will allow for the implementation of prostheses. Although one recent approach, the direct anterior approach for THA, has gained popularity in recent years, its long learning curve creates potential disadvantages and makes the approach challenging [6]. Khan et al. [7] described a minimally invasive posterior approach that preserved the piriformis muscle. The posterior approach is still the most frequently employed surgical technique for THA due to its visual advantages, which enable the joint replacement procedure to be more precise [8].

Implant placement and minimal soft tissue damage are of great importance to prevent complications that may occur in the hip after THA surgery and achieve better functional results [9]. This study aimed to elucidate the radiological and functional results of patients with THA with a piriformis tendon-sparing posterior approach (PSPA) and those with a conventional direct posterior approach (CPA) where the piriformis tendon is cut. In this article, answers were sought to the questions of (i) whether there is an effect on implant dislocation of not cutting the piriformis muscle and (ii) whether there is an effect on early discharge, pain, and hip function. We hypothesized that PSPA would show better results in implant dislocation, hospital stay, pain, and hip functions than CPA.

## 2. Methods

A retrospective evaluation was performed on those who underwent THA with a direct posterior approach, sparing the piriformis muscle between April 2018 and June 2020 at Private Medicabil Hospital. For comparison, the CPA group was examined retrospectively in patients who underwent THA with a direct posterior approach between January 2016 and April 2018. The study was conducted in accordance with the Declaration of Helsinki, and the protocol was approved by the Health Sciences University Bursa High Specialized Education and Research Hospital Ethics Committee (protocol code KAEK-25/03-08, approval date 22 March 2023).

### 2.1. Patients

A G*power (Version 3.1) analysis program determined the study’s sample size. According to the priori power analysis of the *t*-tests based on a functional score of the reference study [10], the priori calculated sample size was at least 300 patients (150 per group) with a power of 90% (d = 0.37) and an *a* level of 0.05. Three-hundred and fifty patients with complete demographic, preoperative, and postoperative data were divided into PSPA (n = 163) and CPA (n = 187) groups. The included patients in both groups had been diagnosed with primary osteoarthritis, avascular necrosis, congenital hip dysplasia, Perthes disease, and femoral neck fracture and had no history of surgery in the operated hip. Patients were excluded from the study if they had a history of surgery in the same hip, bilateral THA, trauma, dementia, excessive external rotation deformity, Crowe type 3 or 4 hip dislocation, body mass index over >40 kg/m^2^, or if they could not cooperate with standard physical therapy and rehabilitation protocols.

### 2.2. Surgical Technique

Our hospital’s orthopedics and traumatology unit is structured in a special arthroplasty team, including a single, well-experienced surgeon, assistant physicians, an anesthesiologist, arthroplasty nurses, and assistant personnel. This multidisciplinary and specialized surgical team has been assigned exclusively to arthroplasty patients for many years, contributing to the standardization of the consistent execution of surgical procedures. This specialized surgical team performed all the operations using uncemented acetabular and femoral components. The patients in both groups received general or regional anesthesia, and all the operations were performed under hypotensive anesthesia.

**Figure 1:**
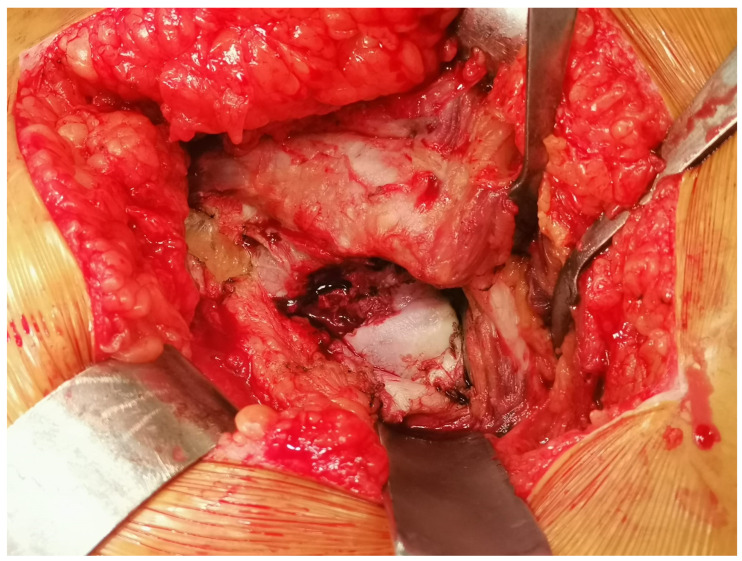
Arthrotomy with preservation of piriformis.

**Figure 2:**
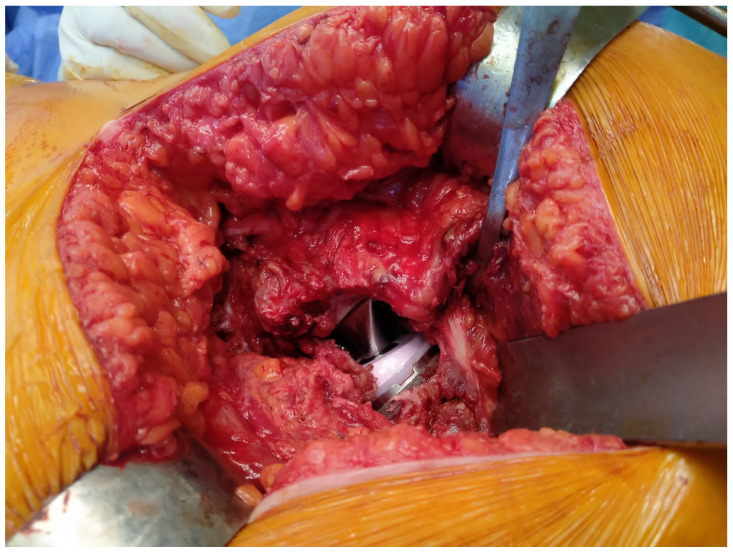
Joint reduction and preserved piriformis tendon after component placement.

*Piriformis-sparing posterior approach:* The external rotators and capsule were cut from the attachment site to the trochanter, preserving the piriformis tendon. Following component placement, the other short external rotators and capsule were sutured to the trochanter with two sutures using the opened holes, as in the conventional posterior approach group. In the PSPA group, if the components were placed at the planned offset, then the tension of the piriformis tendon during traction applied distally in 5–10° flexion and neutral adduction should be observed to cause a reduction in the jumping distance of the head compared to the conventional posterior approach cases where the piriformis tendon was cut. Insufficient or too much tension of the piriformis tendon should suggest that the components are not placed at the appropriate offset (horizontal and vertical). Following hip reduction under anesthesia, checking joint stability in 90° flexion and 45° internal rotation was performed, and an intact endpoint was felt (Figure 1 and Figure 2) [11].

*Conventional posterior approach:* The patients were placed in the lateral decubitus position with the affected side uppermost. Following routine disinfection and draping, entry was made with a direct posterior incision. The trochanteric region was reached by passing through all the layers. The piriformis tendon was located by elevating the posterior edge of the gluteus medius, then cut as close as possible to the piriformis fossa insertion site, preserving the gluteus minimus, which was moved to the posterior. The gluteus minimus was separated from the capsule. Parallel to the femoral neck from the posterior edge of the gluteus minimus, the capsule and other external rotators were cut from the attachment site at the posterior of the trochanter major and retracted to the posterior. The gluteus maximus was released 1.5 cm from the attachment site to the linea aspera to prevent trauma to the sciatic nerve. Following component placement, the piriformis and capsule were sutured with one suture, and the other rotators and capsule with two sutures, to the trochanter major through three holes that had been opened. Suturing was applied to the abduction and external rotation [12].

Both groups were administered low-molecular-weight heparin prophylaxis for deep vein thrombosis and second-generation cephalosporin as surgical infection prophylaxis. Tranexamic acid (IV and local) was administered for bleeding control, and a periprosthetic injection (Ranawat cocktail) was given as analgesia [13].

### 2.3. Rehabilitation Protocol

All patients followed the same standard rehabilitation protocol from postoperative day 0 to the third month postoperatively. The rehabilitation protocol included ankle pumping, isometric setting of quadriceps and hip adductors, in-bed activities, sitting upright, hip range of motion exercises, and walking using a walker as tolerated within 24 h of surgery during hospitalization with a specialized physiotherapist twice a day. All patients were discharged when meeting the criteria for making self-independent daily activities, sitting, and independent mobility using a walker. The standard physical therapy protocol after discharge aimed to train neuromuscular functioning by doing several repetitions of various ambulatory exercises. All the patients were scheduled for physical therapy controls every two weeks to set the daily exercise program, including stair climbing, sit-to-stand, sidestep-up-down, and walking exercises, regarding their functional capability (8–15 repetitions with 1–2 sets or 15 to 30 min).

### 2.4. Outcomes

The demographic data (age, gender, diagnosis, operation side) and the preoperative American Society of Anesthesiologists (ASA) scores of both groups were recorded. The PSPA and CPA groups were compared in terms of anesthesia type, operating time, and hospital stay.

The internal rotation test was applied to evaluate whether the posterior capsule and soft tissues were intact postoperatively. This test was performed six weeks postoperatively, with the patient positioned supine and the hip and knee in 90° flexion. The test was evaluated as positive if an intact endpoint was felt with 15° internal rotation, as described by Ranawat et al. [14] The same orthopedist performed the internal rotation test in both groups.

Hip pain and function assessments were made by an independent physiotherapist preoperatively and at six weeks postoperatively. The Visual Analog Scale (VAS) and the 0–48-point Oxford Hip Score (OHS) were used to evaluate pre- and postoperative pain [15] and hip function [16]. A higher OHS indicates better hip function.

The inclination and anteversion angles of the acetabular component were measured on the sixth-month postoperative anterior–posterior (AP) pelvis radiographs [17].

### 2.5. Statistical Analysis

Data obtained in the study were analyzed statistically using IBM SPSS software (IBM SPSS Statistics for Windows, Version 23.0. Armonk, NY, USA). The conformity of the data to normal distribution was examined with the Shapiro–Wilk test. Descriptive statistics were stated as median (minimum–maximum) values for continuous variables and as a number (n) and percentage (%) for categorical variables. A Student *t*-test or the Mann–Whitney U test was used to analyze score differences between the two groups. The Pearson Chi-Square and Fisher’s Exact tests were used in categorical data analyses. The level of statistical significance was set as *p* < 0.05.

## 3. Results

The demographic analysis showed that the PSPA group comprised 65 males and 98 females with a mean age of 60.86 years and a mean follow-up of 19.1 months. The CPA group comprised 60 males and 127 females with a mean age of 59.17 years and a mean follow-up of 46 months. There was no difference between the groups regarding age and preoperative ASA scores (*p* > 0.05). However, 77 patients were right, and 86 patients were left-side operated in the PSPA group, whereas 109 patients were right, and 78 patients were left-side operated in the CPA group (*p* = 0.039) (Table 1).

The general anesthesia rate was higher in the PSPA group (77.9%) than in the CPA group (61.5%) (*p* = 0.001). The epidural anesthesia rate was higher in the CPA group (38.5%) than in the PSPA group (22.1%) (*p* = 0.001). In the Ranawat internal rotation test, the positivity rate was higher in the PSPA group (n = 146, 89.6%) than in the CPA group (n = 75, 40.1%) (*p* = 0.001). The mean operating time was 58.26 min in the PSPA group and 58.58 min in the CPA group (*p* = 0.256). The length of stay in the hospital was 2.14 days in the PSPA group and 2.47 days in the CPA group, and the difference was statistically significant in favor of the PSPA group (*p* = 0.006), without meaningful differences in a clinical manner. Only 6.1% of patients in the PSPA group and 7.4% in the CPA groups needed blood replacement during the surgery (*p* = 0.618). Dislocation occurred in three (1.6%) patients in the CPA group: one in the sixth postoperative week, one in the tenth week, and one in the fourth month (*p* = 0.104). All the dislocations were treated with closed reduction under anesthesia, and no further dislocation developed during follow-ups (Table 2).

Statistically significant improvements were found in both groups in comparing intra-group preoperative and postoperative concerning the VAS hip pain and OHS (*p* < 0.05). There was no statistical difference between groups in preoperative and sixth-week postoperative VAS hip pain and OHS (*p* > 0.05) (Table 3).

When the sixth-month postoperative anteversion of the acetabular component was examined, there was no statistical difference between groups (*p* = 0.523). The postoperative inclination angle of the acetabular component was statistically higher in the PSPA group than in the CPA group (*p* = 0.001), but this difference had no clinical importance between groups (Table 4). No infection history was determined in any patient included in the study.

## 4. Discussion

The present study clarifies several critical findings in comparing the piriformis-sparing and conventional approaches. The dislocation rate was low, occurring only in the CPA group, as three patients had subsequent dislocations that needed closed reduction; however, no dislocations occurred in the piriformis-sparing approach. Notably, the Ranawat internal rotation test revealed a markedly higher positivity rate in the PSPA group. The mean operating times, hospital stays (clinically), and improvements in VAS hip pain and OHS differences were similar between groups. The postoperative anteversion and inclination angles of the acetabular component were similar in the clinical manner.

The restricting effect of the joint capsule on functional internal rotation is lost when it is cut during opening. Earlier studies reported a postoperative rupture in the external rotators in patients with unrepaired or repaired joint capsules during the closure of most cases [18,19]. With the internal rotation test described by Ranawat et al. [14], the integrity of these structures can be checked early. In cases where the piriformis tendon has been cut or not, the distance of the head from the acetabular component is reduced due to distal traction of the extremity in cases with equal limb length. With the thought that there could be a risk of dislocation in THA cases applied by cutting the piriformis muscle, one of the reasons for dislocation, which is the tendency to keep vertical offset high, will cause limb length discrepancy [20]. In this study, the internal rotation test was positive at the end of the sixth postoperative week in 146 (89.6%) patients in the PSPA group and 75 (40.1%) in the CPA group. The 89.6% positivity rate in the Ranawat internal rotation test in the PSPA group was likely the preserved stability effect of the uncut piriformis on the healing of the capsule and the other external rotators.

The posterior approach is widely used in THA as it provides an excellent visual field, can be applied easily, allows for easy intervention for complications that may develop, and does not damage the abductor mechanism [7,21]. The main disadvantage of the posterior approach is cutting the short external rotator muscles, which play an essential role in hip joint stability [10]. However, little was found in the literature on questioning stability by leaving the piriformis intact in THA surgery. A previous study showed that leaving the piriformis intact reduces the risk of dislocation by preventing excessive internal rotation in such a hip [7]. Moreover, repair of the capsule and other external rotators also prevents rupture by restricting internal rotation [7,22,23]. However, studies have also reported a high rate of early rupture of repaired external rotators [19,20]. Early ruptures were detected in the proportion of 70–80% in the first six weeks in THA patients where only the external rotators and the capsule were not repaired [19]. The unrepaired joint capsule losing the role of restricting internal rotation played a significant part in this failure. A previous systematic analysis reported 1.01% dislocation rates in the posterior approach in the short-term from 6 to 60 months of follow-up [24]. In a hip with soft tissue balance obtained with appropriate vertical and horizontal femoral offset and appropriate anteversion and inclination angles of the acetabular component, dislocation occurs in excessive internal rotation and adduction. Although there was no component malpositioning or soft tissue imbalance in the current study in either group, which would increase the dislocation risk. The results indicated that the dislocation rate was low compared to the literature findings; dislocation occurred in only three patients in the CPA group; however, no dislocations occurred in the piriformis-sparing approach, which indicated sparing the piriformis muscle may have been advantageous from a clinical perspective for short-term outcome in THA patients. However, the low rate of dislocation complications (1.6%) in the CPA group and the fact that this result was similar compared to literature findings indicates that both surgical methods had higher success rates for the dislocation complication.

Reducing surgery-related soft tissue trauma leads to better clinical results in hip arthroplasty [25]. Surgery with reduced soft tissue trauma has been used extremely often in recent years. A previous study compared conventional and piriformis-sparing groups and determined lower pain levels in the piriformis-sparing group, with shorter hospital stays, fewer drug requirements, and earlier independent walking. After discharge, the pain and functional results were similar in both groups [26]. Another finding is that a previous study compared the piriformis-sparing and conventional approaches and showed that the piriformis-sparing approach gained a superior early to mid-term pain and functional outcome than the conventional approach. The current study found that hip pain and OHS improved significantly in both groups’ sixth week after surgery, without superiority. These results may be due to the effectiveness of both surgeries in relieving pain. In addition, the standard postoperative rehabilitation program applied to both groups may have played a significant role in achieving similar functional outcomes. The physical therapy training provided during the early recovery phase focused on helping patients adapt to daily life and regain independence as quickly as possible. This early rehabilitation process likely facilitated functional recovery by promoting patient mobilization, resulting in comparable outcomes for both surgical methods.

Previous reports stated that the mean operating time in the posterior THA approach was a mean of 46–118 min [25,27]. However, in our study, the average surgical time without the duration of anesthesia in both groups was 58 min. This isolated surgical time may suggest that THA surgery utilizing the piriformis-sparing approach is performed at a similar operative time as the conventional posterior approach, possibly leading to lower dislocation rates after surgery. With advanced anesthesia and surgical techniques applied in recent years, early mobilization and hospital stays after THA range from one to three days [28] and four to five days [29]. Previous works report that the length of stay was 3.5 days [21] and 4 days [30] in posterior approach groups. However, the current study revealed that the mean length of stay was 2.14 and 2.47 days in the PSPA and CPA groups, respectively, with the length of stay being statistically longer in the conventional approach without meaningful differences in a clinical manner between groups. Many factors may affect the length of stay in the hospital including the type of anesthesia, need for anesthesia, comorbidities, or the type of analgesics used. Therefore, it is possible to conclude that the results indicate that patients’ length of stay did not differ by the piriformis-sparing or conventional posterior surgical approaches in THA surgery.

Factors affecting joint stability after THA included insufficient limb length discrepancy, malpositioning of components, impingement, pelvic obliquity, and tilt [31]. The target anteversion range for the acetabular cup position was 10 to 30 degrees, and the target inclination range was 30 to 50 degrees [32]. A previous report indicated a ‘’safe zone’’ for acetabular cup inclination of 40 ± 10 degrees and acetabular anteversion of 15 ± 10 degrees to minimize dislocation after THA [33]. At the end of a 24–36-month follow-up period, Kim et al. [34] determined no significant difference in the patient’s radiological results with a standard approach (anteversion angle: 26; inclination angle: 41.9) to THA and those where the piriformis muscle was preserved (anteversion angle: 29; inclination angle: 44.3). In the current study, while the acetabular inclination angle was significantly higher in the PSPA group (36.7°) than in the CPA group (35.3°), the groups had comparable acetabular anteversion angles (PSPA and CPA: 14.2° and 14.3°, respectively). In addition, the fact that the acetabular inclination and anteversion angles are within safe ranges as previously defined [33] in both groups indicates that the piriformis-sparing approach we applied is successful in maintaining its continuity in the short term, nearly 1.5–2 years after the piriformis-sparing group, and nearly 3.5–4.5 years after the conventional approach group.

Upon reviewing the existing research, it can be suggested that future studies should emphasize the long-term outcomes of piriformis-sparing versus conventional posterior surgical approaches. Key areas for investigation should include dislocation rates, joint stability over time, implant survival, and the relationship between joint capsule integrity and functional outcomes in the piriformis-sparing technique. Additionally, comparisons of structured physiotherapy programs are essential to understand their impact on recovery, particularly the effects of late postoperative interventions. Furthermore, incorporating patient-reported outcomes, quality-of-life assessments, and satisfaction questionnaires will provide valuable insights into the overall success of both surgical methods.

This study has several limitations. First, the fact that we applied the Ranawat internal rotation test only to the extremity that underwent THA may have affected the results of this test. We did not question hip pain or functional status at the last follow-up in our study. In addition, the groups had different follow-up periods, and the duration of anesthesia could not be included in the total surgical time because it was not recorded in the hospital records. Since implant dislocation is a multifactorial issue, other external factors, including patient awareness and educational level, may have affected the dislocation outcomes in the study. Lastly, the data on spinopelvic parameters were not included. The evaluation of spinopelvic parameters and acetabular component placement associated with these parameters could have played an essential role in determining the risk of dislocation. The influence of spinopelvic angles may directly affect implant placement and joint stability, which may affect surgical outcomes.

## 5. Conclusions

This study hypothesized that the piriformis-sparing posterior approach would show better results in implant dislocation, hospital stay, pain, and hip functions than the conventional posterior approach. The hypothesis regarding dislocation rates and functional outcomes was not confirmed in light of the outcomes obtained. Based on these results, we recommend that surgeons consider utilizing both surgical approaches for THA patients. However, the absence of dislocation cases in the piriformis-sparing approach suggests that preservation of the piriformis tendon, especially in the early period, may have reduced the risk of prosthesis dislocation by increasing joint stability from a clinical perspective. A larger sample size and detailed multicenter follow-ups further enhance the applicability of these findings in clinical practice. Further prospective randomized controlled studies are needed to assess the long-lasting impact of the piriformis-sparing posterior approach regarding the dislocation rates and functional outcomes.

## Figures and Tables

**Table 1:** Demographic data of the groups at baseline.

	Piriformis-Sparing Posterior Approach	Conventional Posterior Approach	
	(n = 163)	(n = 187)	
	Mean (Min–Max)	Mean (Min–Max)	*p* ^1^
Age (years)	60.86 (26–88)	59.17 (24–87)	0.142
	n (%)	n (%)	Total
Gender			
Male	65 (40%)	60 (32%)	125 (35.7%)
Female	98 (60%)	127(68%)	225 (64.3%)
Total	163 (100%)	187 (100%)	350 (100%)
Preoperative diagnosis			
Primary osteoarthritis	43 (26.4%)	63 (33.7%)	106 (30.3%)
Avascular necrosis	26 (16%)	20 (10.7%)	46 (13.1%)
Congenital hip dysplasia	46 (28.2%)	53 (28.3%)	99 (28.3%)
Perthes disease	10 (6.1%)	8 (4.3%)	18 (5.1%)
Femoral neck fracture	38 (23.3%)	43 (23%)	81 (23.2%)
Total	163 (100%)	187 (100%)	350 (100%)
Operated side			*p* ^2^
Right	77 (47.2%)	109 (58.2%)	0.039 *
Left	86 (52.8%)	78 (41.8%)	
Total	163 (100%)	187 (100%)	
ASA score			
1	73 (44.8%)	80 (42.8%)	0.121
2	70 (42.9%)	82 (43.8%)	0.098
3	18 (11.1%)	23 (12.3%)	0.102
4	2 (1.2%)	2 (1.1%)	0.870
Total	163 (100%)	187 (100%)	

Min: minimum; Max: maximum; ASA: American Society of Anesthesiology; *p*^1^: Mann–Whitney U test; *p*^2^: Pearson’s Chi-Square test and Fisher’s Exact test. Significant *p*-values are marked with *.

**Table 2:** Comparison of the clinical data between groups.

	Piriformis-Sparing Posterior Approach	Conventional Posterior Approach	
	(n = 163)	(n = 187)	
	n (%)	n (%)	*p* ^1^
Type of anesthesia			
General	127 (77.9%)	115 (61.5%)	0.001 *
Epidural	36 (22.1%)	72 (38.5%)	0.001 *
Sixth-week positive Ranawat internal rotation test	146 (89.6%)	75 (40.1%)	0.001 *
Dislocation complication	0 (0%)	3 (1.6%)	0.104
Blood replacement	10 (6.1%)	14 (7.4%)	0.618
	Mean (Min–Max)	Mean (Min–Max)	*p* ^2^
Operation time (minute)	58.26 (50–65)	58.58 (50–65)	0.256
Hospital stays (day)	2.14 (1–5)	2.47 (1–8)	0.006 *

Min: Minimum; Max: Maximum; *p*^1^: Pearson’s Chi-Square test and Fisher’s Exact test; *p*^2^: Mann–Whitney U test. Significant *p*-values are marked with *.

**Table 3:** Comparisons of pain and function scores at baseline and after the surgery between groups.

	Baseline (Before the Surgery)		After Surgery (6 Weeks Postoperatively)		
	Mean	Min–Max	Mean	Min–Max	*p^b^*
VAS (cm)					
Piriformis-Sparing Posterior Approach	6.35	2–10	2.92	0–6	0.001 *
Conventional Posterior Approach	6.40	4–10	2.98	2–5	0.001 *
*p^a^*	0.448		0.734		
OHS					
Piriformis-Sparing Posterior Approach	11.91	4–23	40.40	22–48	0.001 *
Conventional Posterior Approach	11.33	2–18	40.08	26–46	0.001 *
*p^a^*	0.080		0.776		

VAS: Visual Analogue Scale; OHS: Oxford Hip Score; Min: minimum; Max: maximum; *p^a^*: Mann–Whitney U test, value of between-group comparison analyses; *p^b^*: Mann–Whitney U test, value of within-group comparison analyses. Significant *p*-values are marked with *.

**Table 4:** Postoperative sixth-month component alignment comparisons between groups.

	Piriformis-Sparing Posterior Approach (n = 163)	Conventional Posterior Approach (n = 187)	*p*
	Mean (Min–Max)	Mean (Min–Max)	
Acetabular component anteversion angle (degrees)	14.22 (8–25)	14.37 (8–25)	0.523
Acetabular component inclination angle (degrees)	36.78 (25–45)	35.36 (26–52)	0.001 *

Min: minimum; Max: maximum; *p*: Student *t*-test; Significant *p*-values are marked with *.

## Data Availability

Due to privacy and ethical restrictions, the data are not publicly available.
